# Design and Evaluation of PCR Primers for Denaturing Gradient Gel Electrophoresis Analysis of Plant Parasitic and Fungivorous Nematode Communities

**DOI:** 10.1264/jsme2.ME12158

**Published:** 2013-04-17

**Authors:** Atsuhiko Kushida

**Affiliations:** 1 Upland Farming Research Division, NARO Hokkaido Agricultural Research Center, Shinsei-minami 9–4, Memuro-cho, Kasai-gun, Hokkaido 082–0071, Japan

**Keywords:** detection sensitivity, fungivorous nematode, order *Tylenchida*, PCR-DGGE primer, plant parasitic nematode

## Abstract

A PCR-DGGE primer pair, Tyl2F-Tyl4R, specific to plant parasitic and fungivorous nematodes was designed based on the 18S rRNA gene. The results of community analysis using the primers showed that they are specific to the order *Tylenchida*. This primer pair detected species belonging to *Tylenchida* with high sensitivity and high resolution. The number of detected species of plant parasitic and fungivorous nematodes and their band intensity were much improved compared with PCR-DGGE analysis using the SSU18A-SSU9R primer, which is commonly used for nematode community analysis. It was confirmed that using a group-specific primer was effective for nematode community analysis with PCR-DGGE.

A diverse range of nematodes live in all soil ecosystems. They have important roles in soil function, the cycling of plant nutrients and decomposition of organic matter, and are highly sensitive to various environmental factors such as fertilization and plant cultivation (5, 12, 15, 20). Therefore, the analysis of soil nematode communities has evolved as a bioindicator for the evaluation of soil function and the attributes of the soil food web (4, 6, 10, 21).

Although nematode community analysis has mostly been conducted based on the morphological identification of individual specimens, this analysis is laborious and time consuming, and requires specific skills and experience for accurate nematode identification. To solve these problems, attempts have been made to analyze nematode communities using molecular biology techniques to detect differences in the DNA sequences of individual species, and polymerase chain reaction–denaturing gradient gel electrophoresis (PCR-DGGE) has attracted the most attention and use (2, 6, 7, 13, 19). PCR-DGGE detects different nematode taxa as different bands of PCR products that are denatured depending on their DNA sequence on a polyacrylamide gel with a concentration gradient of the denaturant, which is a mixture of urea and formamide. The advantage of PCR-DGGE analysis is that nematode taxa corresponding to each band can be identified by determining the sequence of the DNA and comparing that with a DNA database.

The author has investigated many nematode community structures in field soils using the PCR-DGGE method developed by the National Institute for Agro-Environmental Sciences (NIAES) (http://www.niaes.affrc.go.jp/project/edna/edna_jp/manual_nematode_e.pdf, hereinafter NIAES procedure). This method allows the easy and rapid examination of a number of nematode communities as compared with morphological identification. However, some researchers have reported that PCR-DGGE lacks detection sensitivity when the nematode community being assessed consists of a large number of species (2, 6). The author also has firsthand experience of this insufficient sensitivity.

It is particularly important to be aware of the existence of plant parasitic nematodes when analyzing field nematode communities because they are considered one of the most important bioindicators of soil condition and are therefore important for the cultivation of healthy crops; however, many plant parasitic species are difficult to detect with PCR-DGGE analysis because they are present in insufficient quantities. As fungivorous nematodes have close interaction with fungus fauna and respond to various agricultural management sensitively (22), it is important to assess them to understand the function of nematode fauna in detail; however, since many fungivorous species tend to present in a small number in many fields (A. Kushida, unpublished results), they are hard to detect with PCR-DGGE.

Using group-specific primer sets in PCR-DGGE analysis increased the technique’s sensitivity to detect target bacteria in a bacterial community (3, 9). It was therefore hypothesized that also in a nematode community, the use of group-specific primer pairs in PCR-DGGE analysis may allow the sensitive detection of plant parasitic and fungivorous nematodes even in a complex nematode community. Plant parasitic and fungivorous nematodes have a common morphological characteristic of having a ‘stylet’ in their head. The stylet is a vital organ for feeding and is the most important basis to distinguish these nematodes. Since they are classified into similar taxa based on this common characteristic, it was expected that these nematodes can be grouped. Meanwhile, there are also a few plant parasitic and fungivorous species in a taxonomic group that do not have a stylet. They have a ‘odontostyle’ that resembles a stylet and belong to order *Dorylaimida*. Since order *Dorylaimida* locates to a distant taxon from the group with a stylet, it was thought that designing a primer that could also target *Dorylaimida* would be very difficult.

The objective of this study was to design primers that specifically amplified the DNA of principal species of plant parasitic and fungivorous nematodes with a stylet, and to evaluate the efficiency of PCR-DGGE analysis of nematode communities.

18S rRNA gene sequences of nematode species (Table 1) were obtained from the DNA Data Bank of Japan (DDBJ) database and compared using DNASIS PRO (Hitachi Solutions, Shinagawa, Japan). Highly specific regions of the RNA gene for plant parasitic and fungivorous nematodes were selected, and primers were designed for those regions. The four plant parasitic nematode species used for sequence comparison are distributed in some or most of the fields in Hokkaido, Japan, and the two fungivorous and three bacteriovorous species were also common in these fields from the results of previous DGGE analyses. To evaluate the efficiency of the specific primers, DGGE and excised band sequence analyses were conducted for four nematode communities and the results compared with those of analyses using the primer pair SSU18A (5′-AAAGATTAAGCCAT GCATG-3′) and SSU9R (5′-AGCTGGAATTACCGCGGC TG-3′). This primer pair was designed by Blaxter *et al.* (1) and is commonly used for analyzing soil nematode communities in Japan.

Soil samples were collected from four fields, each located in a different town (Memuro, Kuriyama, Shimizu, and Otofuke) in Hokkaido, Japan. The soil samples were passed through a sieve with a pore size of approximately 5 mm to remove gravel and plant roots. The nematodes were extracted from 20 g soil and nematode DNA was prepared according to the NIAES procedure with one modification: in this study, DNA was extracted from approximately 500 individuals instead of 300 individuals.

Nematode DNA was amplified using two pairs of primers. One primer pair was SSU18A and SSU9R. PCR using this primer pair and purification of the PCR products were also conducted according to the NIAES procedure. Another primer pair was designed and selected in this study. A GC clamp (5′-CGCCCGCCGCGCCCCGCGCCCGGCCCGCCGCCCCCGCCCG-3′) was attached to the 5′ end of the forward primer and then PCR was performed in a 25 μL reaction mixture containing 1 unit of Takara Ex Taq HS polymerase (Takara, Ohtsu, Japan), 1×PCR buffer, 0.2 mM dNTPs, 0.5 μM of each primer, and 2.5 μL nematode DNA solution. The PCR was carried out in a Takara PCR Thermal Cycler Dice mini using the following thermal cycles: an initial step at 95°C for 3 min was followed by 30 cycles of denaturation at 95°C for 30 s, annealing at 52°C for 30 s, and extension at 72°C for 1 min. The final extension step was at 72°C for 7 min.

DGGE was performed using the DCode System (Bio-Rad Laboratories, Hercules, CA, USA) and carried out according to the methods described by Okada and Oba (13) with a few modifications. The denaturant gradient ranged from 22.5% to 40.0% (100% denaturant consisted of 7 M urea and 40% [v/v] formamide). Five microliters of each PCR product were applied to the DGGE gel, and electrophoresis was performed in 1×Tris-Acetate-EDTA (TAE) buffer at 60°C at a constant voltage of 75 V for 16 h. Subsequently, the gel was stained with SYBR Gold nucleic acid gel stain (1:10,000 dilution; Cambrex, Rockland, ME, USA) for 45 min at room temperature.

To define the nematode taxon equivalent to each DGGE band, as many bands as possible were cut out from the gels and placed in collection tubes. After rinsing twice with 50 μL of 10-fold diluted Tris-EDTA buffer (1/10 TE), 20 μL of 1/10 TE was added to each collection tube. The tubes were stored at −20°C and incubated at 95°C for 10 min before use. An aliquot (ca. 2 μL) was used in the PCR with the same primer pair used for DGGE. Takara Ex Taq HS polymerase was used in this PCR reaction. PCR products were treated with ExoSAP-IT (USB, Cleveland, OH, USA) according to the manufacturer’s instructions and were reacted using a BigDye Terminator v3.1 cycle sequencing kit (Applied Biosystems, Carlsbad, CA, USA). Sequences were analyzed on an ABI PRISM 3130-Genetic Analyzer (Applied Biosystems). The determined sequences were submitted to the DDBJ and examined for their taxonomic affinities with BLAST analysis. The name and accession number of the nematode species showing the highest homology value has been listed.

In order to evaluate whether the designed primer could detect all target species in the nematode community, the 18S rRNA gene sequences of most fungivorous nematodes in a community were determined individually, the closest relatives defined, and compared with the result of DGGE analysis using the designed primer. A nematode community from Memuro field was used in this test. According to microscopic observation, the number of fungivorous nematodes per 500 nematodes of the community was 56. Thirty were randomly and individually picked up under a binocular, and each nematode DNA was extracted according to the method described by Uehara *et al.* (18) and amplified using the SSU18A-SSU9R primer pair and then directly sequenced. Sequences were sent to DDBJ and homology was analyzed using BLAST.

Three forward and five reverse primers were designed and used for PCR (Table 2). Although a band was observed on agarose gel with all of the primer pairs used in this study, sharp bands were not observed on DGGE profiles with most primer pairs. Only one pair of primers, Tyl2F (5′-TGGC CACTACGTCTAAGGAT-3′) and Tyl4R (5′-CCCGTTTGT CTCTGTTAACC-3′), produced sharp bands on the DGGE gel even when a GC clamp was attached to the 5′ end of Tyl2F. The nucleotide sequences of primer positions of the species used for sequence comparison are shown in Table 1. Although the nucleotide sequences of Tyl2F were common to all compared plant parasitic and fungivorous nematodes, there were variations at 5 or 6 bases in the corresponding sequences of three bacteriovorous nematodes. Tyl2F’s position is 345 bp behind SSU18A on the 18S rRNA gene sequence of *Pratylenchus penetrans*, and Tyl4R’s position is 272 bp behind SSU9R. The size of the PCR product was 482 bp.

DGGE profiles of the nematode communities in the soils of the four fields analyzed that were obtained using two primer pairs (SSU18A-SSU9R [SSU] and Tyl2F-Tyl4R [TYL]) are shown in Fig. 1. The results of a homology search for the sequences of the DGGE bands in Fig. 1 are shown in Table 3. Several bands resulted from the same nematode species. The author often observed that a single nematode produced multiple bands, and similar phenomena were also observed for a single laboratory-isolated bacteria (11, 16). This may be derived from a variation in their rRNA repeat sequences.

Recently, many PCR-DGGE analyses of soil nematode communities using the primer pair SSU have been performed in Japan and their usefulness has been confirmed (13). However, the number of nematode species detected from the DGGE profile using the SSU primer pair was limited, and bands due to organisms other than nematodes (protozoa, tardigrades, *etc*.) were also observed (Fig. 1 and Table 3). This suggests that SSU is not suitable for the detection of nematode species that are present in only small numbers. On the other hand, only plant parasitic or fungivorous nematodes were detected using TYL, indicating that this primer pair is specific to a certain group of nematodes. SSU could not detect most of the nematode species detected by TYL, and the band intensities with SSU were weaker than those with TYL, even when bands derived from the same species were detected by both primer pairs. Species that were undetectable with SSU could still not be detected even when much more amplified DNA was applied to the DGGE gels. These results suggest that TYL is more suited to the detection of plant parasitic and fungivorous nematodes than SSU.

BLAST analyses of 18S rRNA gene sequences which were individually determined for 30 fungivorous nematodes from the Memuro field revealed five closest fungivorous species (Table 4). In the DGGE analysis, TYL detected three fungivorous species, *Filenchus misellus*, *Ditylenchus* sp., and *Pseudhalenchus minutus*, from same community, but could not detect *Aphelenchoides* sp. and *Laimaphelenchus heidelbergi* (Table 3). This suggests that TYL does not target all fungivorous species with a stylet. All plant parasitic and fungivorous nematodes with a stylet are classified into one of two orders: *Tylenchida* (17) or *Aphelenchida* (8). *F. misellus*, *Ditylenchus* sp. and *P. minutus* belong to order *Tylenchida*, and *Aphelenchoides* sp. and *L. heidelbergi* belong to order *Aphelenchida*. Moreover, every plant parasitic and fungivorous nematode detected in the four nematode communities by TYL belongs to *Tylenchida* (Table 3). Then, 18S rRNA gene sequences of important plant parasitic and fungivorous nematodes belonging to *Tylenchida* and *Aphelenchida*, *Tylenchorhynchus dubius* (EU306352), *Meloidogyne incognita* (AY284621), *Rotylenchus uniformis* (AY593882), *Helicotylenchus dihystera* (AJ966486), *Aphelenchoides besseyi* (AY508035), and *Aphelenchus avenae* (JQ348399), were also derived from the DDBJ database and surveyed. Although every species belonging to *Tylenchida* had the same sequences as Tyl2F and Tyl4R, variation was observed in four or five bases of the sequence corresponding to Tyl2F of the species belonging to *Aphelenchida* (*Aphelenchoides besseyi* and *Aphelenchus avenae*). These results indicate that these primers appear to select for the order *Tylenchida*. Most plant parasitic species belong to *Tylenchida*, and the principal plant parasitic nematode species in fields in Japan, root lesion nematodes, root knot nematodes, and cyst nematodes, also belong to *Tylenchida*. Since most fungivorous species in the fields also belong to *Tylenchida*, it is useful to understand the characteristics of the nematode fauna in the field when analyzing the *Tylenchida* community. As *Aphelenchus* spp. and *Aphelenchoides* spp. are also important fungivorous nematode species, primers designed to target these nematode species will be needed to analyze overall fungivorous communities.

Three *Tylenchida* species, *Filenchus misellus*, *Ditylenchus* sp., and *Pseudhalenchus minutes*, which were detected by individual sequence analysis, were also detected by DGGE analysis using TYL. Therefore, DGGE analysis using TYL may be sensitive for the detection of fungivorous *Tylenchida* species in a community. It will be necessary to confirm its effectiveness using more nematode communities.

TYL was also good for evaluating plant parasitic nematodes. Although root lesion nematodes (*Pratylenchus* sp.) were observed in every tested community through microscopy, SSU failed to detect them in the communities from Shimizu and Otofuke fields, probably due to their small numbers. TYL, however, could clearly detect them. Moreover, since TYL was able to detect a single individual of *P. penetrans*, which was added to a nematode community that consisted of 2,000 individuals without a *Pratylenchus* sp. (data not shown), the detection sensitivity of DGGE using TYL was confirmed to be very high.

Furthermore, DGGE analysis using TYL could clearly detect two *Pratylenchus* spp. (*P. penetrans* and *P. crenatus*) that coexisted in the communities from Kuriyama and Otofuke fields (Table 2). Coexistence of these *Pratylenchus* spp. was also confirmed by PCR-RFLP analyses that were conducted for ten individuals of root lesion nematodes according to the method described by Orui and Mizukubo (14). Although there are many fields where plural plant parasitic species belonging to the same genus coexist, it is very difficult to find this coexistence and identify them by morphological investigation or conventional molecular biological techniques. In many cases, only the dominant species can be identified; however, DGGE analysis using TYL enabled immediate detection with high resolution. These results suggest that DGGE analysis using TYL will become a powerful tool for diagnosing plant parasitic nematodes in the field.

In this study, it was confirmed that the sensitivity of DGGE analysis is improved by the use of group-specific primers; therefore, the usefulness of DGGE analysis is expected to increase through the practical use of such primers.

The determined nucleotide sequences in this study have been submitted to the DDBJ database with accession numbers AB731478–AB731558 (sequences of DGGE bands), AB731159–AB731185 (sequences of fungivorous nematodes from the Memuro field).

## Figures and Tables

**Fig. 1:**
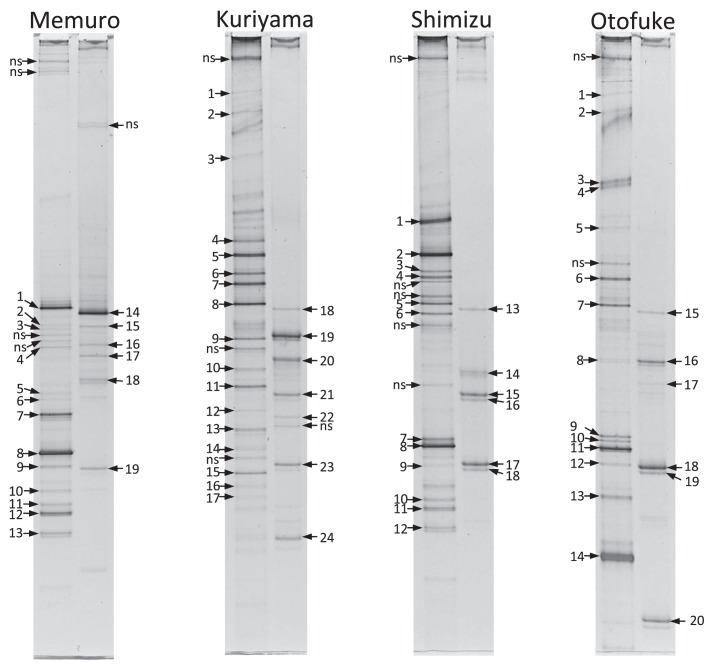
DGGE profiles of the four nematode communities obtained using two primer pairs. DGGE profiles obtained with the primer pair SSU18A-SSU9R are shown in the left lane and profiles obtained with Tyl2F-Tyl4R are shown in the right lane for each nematode community. Numbers indicate the DGGE bands for sequence analysis and homology searches. These numbers are also shown in Table 2. Bands marked ‘ns’ could not be sequenced.

**Table 1:** Nematode species and accession numbers of the 18S rRNA gene sequences used for comparison, and their sequences of designed primer position.

Nematode	Accession No.	Feeding habits^a^	Sequence of primer position (5′ to 3′)	

			Tyl2F	Tyl4R
*Pratylenchus penetrans*	EU669926	pp	TGGCCACTACGTCTAAGGAT	CCCGTTTGTCTCTGTTAACC
*Pratylenchus crenatus*	AY284610	pp	TGGCCACTACGTCTAAGGAT	CCCGTTTGTCTCTGTTAACC
*Heterodera glycines*	AY043247	pp	TGGCCACTACGTCTAAGGAT	CCCGTTTGTCTCTGTTAACC
*Ditylenchus destructor*	AY593912	pp, f	TGGCCACTACGTCTAAGGAT	CCCGTTTGTCTCTATTAACC
*Filenchus misellus*	AB473564	f	TGGCCACTACGTCTAAGGAT	CCCGTTTGTCTCTGTTAACC
*Pseudhalenchus minutus*	AY593916	f	TGGCCACTACGTCTAAGGAT	CCCGTTTGTCTCTATTAACC
*Acrobeloides apiculatus*	AY284673	b	TCGCGAACACATCTAAGGAA	CCCGTTTGTCCCTGTTAACC
*Rhabditis* sp.	EU196004	b	CGGCTACCACATCCAAGGAA	CCCGATTTGTCTCTCTTAATC
*Pristionchus lheritieri*	FJ040439	b	CGGCTATCACATCCAAGGAA	CCCGAGTTGTTCCTTTTAATC

a pp: plant parasitic, f: fungivorous, b: bacteriovorous

**Table 2:** Seqences of designed primers

Primer name	Sequence (5′ to 3′)
Tyl1F^a^	ATCCTTAGACGTAGTGGCCA
Tyl2F	TGGCCACTACGTCTAAGGAT
Tyl3F	CAGAATAACTMAGCTGATCG
Tyl1R	CTACCATCGAAAGTTGATAA
Tyl2R	GCCTGCTGCCATCCTTAGAC
Tyl3R	CCGCAGCAATAATTCTATGCAT
Tyl4R	CCCGTTTGTCTCTGTTAACC
Tyl5R	CCAAGAATTTCACCTCTCACGT

a F: forward primer, R: reverse primer

**Table 3:** Comparison of species detected by DNA sequences of bands amplified by PCR-DGGE with two different primer pairs

Closest species^b^ (order)	Accession No.	Memuro				Kuriyama				Shimizu				Otofuke			
			
		SSU^a^		Tyl^a^		SSU		Tyl		SSU		Tyl		SSU		Tyl	
							
		Band^c^ No.	Highest similarity (%)	Band No.	Highest similarity (%)	Band No.	Highest similarity (%)	Band No.	Highest similarity (%)	Band No.	Highest similarity (%)	Band No.	Highest similarity (%)	Band No.	Highest similarity (%)	Band No.	Highest similarity (%)
Plant parasitic nematode																	
*Heterodera glycines* (Tylenchida)	AY043247													13	99.6	20	99.8
*Paratylenchus dianthus* (Tylenchida)	AJ966496							22	97.4								
*Pratylenchus crenatus* (Tylenchida)	EU130800							20	98.1							16, 17	99.8
*Pratylenchus penetrans* (Tylenchida)	EU130803	5, 6, 7	100	14, 15, 16	99.3	12	100	18	99.8			13	99.3			15	99.3

Fungivorous nematode																	
*Aphelenchoides* sp. (Aphelenchida)	EU287589	10	97.1														
*Ditylenchus* sp. (Tylenchida)	AY284637			18	98.9												
*Filenchus discrepans* (Tylenchida)	AB473565							21	99.0	9	98.2	15, 16	99.0				
*Filenchus misellus* (Tylenchida)	AB473564			19	97.8			23	97.4			17, 18	97.4			18, 19	97.9
*Malenchus andrassyi* (Tylenchida)	AY284587											14	91.7				
*Pseudhalenchus minutus* (Tylenchida)	AY593916			17	98.2												
*Tylenchus arcuatus* (Tylenchida)	EU306348					9	99.2	19	97.6								
*Tylenchus davainei* (Tylenchida)	AY284588							24	86.9								

Other nematode																	
*Acrobeles complexus* (Rhabditida)	AY284671	4	96.8														
*Acrobeloides apiculatus* (Rhabditida)	AY284673	1, 2, 3	100			1, 8	100			3, 5, 6	100			7	100		
*Alaimus* sp. (Enoplida)	FJ040489					17	96.6										
*Angiostoma limacis* (Rhabditida)	EU573705					15	79.2										
*Aporcelaimellus obtusicaudatus* (Dorylaimida)	AY284811	9	100														
*Cephaloboides nidrosiensis* (Rhabditida)	AB376908	11, 12, 13	95.3							10, 11, 12	95.2						
*Microdorylaimus* sp. (Dorylaimida)	AJ966492					13	99.7										
*Oscheius* sp. (Rhabditida)	HQ130503					11	99.8										
*Paraguimperia africana* (Ascaridida)	JF803925					2, 7, 10	92.7										
*Parasitorhabditis obtusa* (Rhabditida)	EU003189									4	92.4			6	91.4		
*Pristionchus lheritieri* (Rhabditida)	EU196020									7, 8	97.7			11	97.7		
*Rhabditis* sp. (Rhabditida)	HQ130504	8	100			14	100							8, 9, 10	99.8		

Not nematode																	
*Achlya bisexualis*	DQ403200					4	99.8										
*Enchelys polynucleata*	DQ411861													1	100		
*Enchytraeus christenseni*	GU901873													14	100		
*Halteria grandinella*	AF194410									1	99.4						
*Lagenidium* sp.	HQ384417					6	100										
*Oikomonas* sp.	AY520451					5	99.4			2	99.4						
*Orchitophryidae*	EF023919													3	100		
Phaseoleae environmental sample	EF024946					16	99.2										
*Sterkiella cavicola*	GU942565													2, 4, 5	99.8		
Tardigrada environmental sample	GQ922229													12	99.4		
Uncultured alveolate	HM799981					3	97.9										

a SSU: primer pair SSU18A-SSU9R; TYL: primer pair Tyl2F-Tyl4R

b These organisms had the highest similarities in the BLAST search.

c Band numbers are shown in Fig. 1.

**Table 4:** Closest nematode species to the fungivorous nematodes present in the Memuro field

Closest species (belonging order)	Accession number	Number of nematodes^a^
*Aphelenchoides* sp. (Aphelenchida)	EU287589	14
*Laimaphelenchus heidelbergi* (Aphelenchida)	EU287587	2
*Filenchus misellus* (Tylenchida)	AB473564	3
*Ditylenchus* sp. (Tylenchida)	AY284637	4
*Pseudhalenchus minutus* (Tylenchida)	AY593916	4
Not sequenced		3

a Numbers indicate the number of nematodes corresponding to the species.
